# Technical considerations and functional results in primary uncemented hip arthroplasty using short femoral stems through mini-invasive techniques

**Published:** 2014-09-25

**Authors:** M Moga, ME Pogarasteanu

**Affiliations:** Orthopedics – Traumathology Clinic, "Dr. Carol Davila" Central Military University Emergency Hospital, Bucharest, Romania

**Keywords:** hip arthroplasty, short femoral stem, mini-invasive technique

## Abstract

Abstract

Primary hip arthroplasty is a surgical procedure through which the coxofemoral joint is replaced with a prosthetic implant. Arthroplasties can be total or partial, cemented or uncemented. These procedures are generally indicated as a form of treatment for arthritic pain or in the case of severe trauma, such as femoral neck fractures. The most commonly used approaches are: Smith Peterson, Watson Jones, Hardinge, Moore Southern and Ludloff. Recently, mini-invasive approaches have started being used, while correlated with short femoral stems. Short metaphyseal femoral stems have been introduced as an alternative to conventional stems, having a series of advantages: preservation of bone stock (high cervical osteotomies), preservation of the anatomical anteversion of the femural neck, decrease in cortical stress forces, decrease in the remaining thigh pain, a longer life of the prosthesis, with the possibility of revision to a conventional prosthesis, and the possibility to be used in correlation with mini-invasive procedures. Short femoral stems implanted through a mini-invasive approach allow the conservation of the femoral bone stock, permitting an ulterior re-intervention, in the context of an ageing population, with a globally rising long-term survival rate. Moreover, the superiority of the total hip arthroplasty with a short femoral stem was discussed through mini-invasive approaches, in the day-to-day realities of our Clinic.

## Introduction

Primary hip arthroplasty is a surgical procedure, in which the coxofemoral joint is replaced with a prosthetic implant, as a treatment for arthritic pain, avascular femoral head osteonecrosis, or in the case of severe trauma, like femoral neck fracture. 

 During the last three decades, the total hip arthroplasty procedure has undergone a continuous process of development, becoming one of the best understood and cost-efficient procedures in orthopedic surgery. 

 The post operatory results are remarkable with any of the approaches: anterolateral, lateral, posterolateral, posterior or medial. These are typically made with an incision of 25 to 40 centimeters in length. The survival rate of this procedure is close to 100%. 

 Despite the classical total hip arthroplasty, the procedure’s established success, technical evolution and high patient demands have lead to a need of exploring new possibilities of improving the procedure. Pain and functional deficit tend not to be the only clear indications for surgery, while ever younger patients resort to hip arthroplasty in order to regain an active life. 

 Conservatory surgery aims both at protecting soft tissues and at conserving bone capital; if we cannot be confident that the expected functional life of the prosthesis is greater than the patient’s life expectancy, it is absolutely necessary to consider successive interventions, thus attempting to preserve a maximum of bone stock, by resecting as little at the time of surgery and by optimizing the physiological load in the proximal femur, in order to preserve bone capital on long term. The most adequate prostheses in this sense are the ones with a short femoral stem, having a metaphyseal support. 

 Short femoral stems, also called metaphyseal stems, have been introduced in the surgical practice as an alternative to conventional femoral stems in the primary uncemented total hip arthroplasty as they offer a number of advantages [**1,2**]. 

 Short femoral stems are a current-day approach to dilemma concerning the need to preserve bone stock in total hip arthroplasty, with regard to a possible reintervention, with an ageing target population, an increase in the mean long-term global survival rate. 

 Biomechanical studies have shown that most short femoral stem prostheses (i.e. PROXIMA) do not function according to the "fit and fill" principle, thus providing an excellent axial and rotational stability within the spongious bone, in which the prosthesis lies "suspended" and with which it moves in unison. This reduces the rotational stress within the fixation interface and optimizes the transfer of forces at the metaphyseal level [**3-5**]. A study by Jakubowitz et al. shows that there is no high periprosthetic fracture risk involved in using short stem femural prosthesis, compared to a standard cementless prosthesis, but that rather the risk is dependent on the patient’s body mass index, in the context of a fall [**6**].

**Fig. 1:**
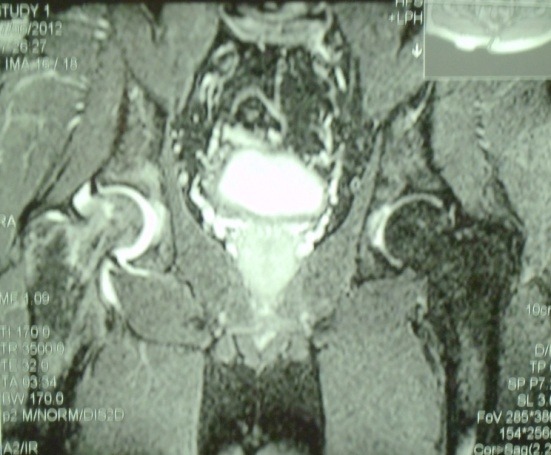
Avascular Necrosis in the right femoral head of a 23-year-old patient, MRI

 Short stem prostheses are designed by the manufacturers to be used in correlation with a mi-neck resection line, preserving bone in the neck and diaphysis area [**7**]. This extra portion of femoral neck also maintains a structural integrity in the femur, adding to the long-term stability, and giving an added degree of torsional stability, crucial in an active, young patient [8,9]. This is also enhanced by the general "fit and fill" design of the short stem prosthesis. The technique of implantation is generally the "round the corner" technique, curving to follow the medial cortical wall, thus preventing the stem from exiting through the lateral wall, white preserving bone mass and providing a favorable angle of approach to the femoral canal. 

 The primary cementless fixation in the femur is done through "scratch fit", which creates a very high coefficient of friction, while long-term fixation is accomplished through osteoconduction. Some short stem prostheses (MiniHip) have anti-rotational fins to add stability in the torsional plane, thus keeping micromotions to a minimum. 

 Considering that most, if not all the patients operated with the short femoral stem are young and active, their high activity levels demand a high range of motion; this is accomplished through the low profile neck and taper of the prosthesis, thus maximizing the head/neck ratio and the safe range of motion [10-13]. 

 The femoral offset also has to be taken into consideration when conducting a hip replacement procedure and short femoral stem prosthetics offers a varying offset and neck lengths, thus allowing a customization of the prosthesis to each patient’s characteristics. 

 Densitometric studies have also shown bone remodeling at the proximal femur following preferentially metaphisary loading in short femoral stem prosthesis [**14**]. 

 As new minimally invasive surgical techniques are explored in hip arthroplasty, we gain a progressive understanding of the fact that, in most cases, "mini-invasive" is associated with a decrease in morbidity, a faster recovery and a more attractive cosmetic result [**15,16**]. 

 In order to facilitate the mini-invasive procedure, a dedicated set of instruments has been developed; including a set of retractors which help achieve an adequate exposure, while at the same time limiting the potential to damage soft tissues, ligaments, or vascular and nervous structures [**17**]. 

**Fig. 2:**
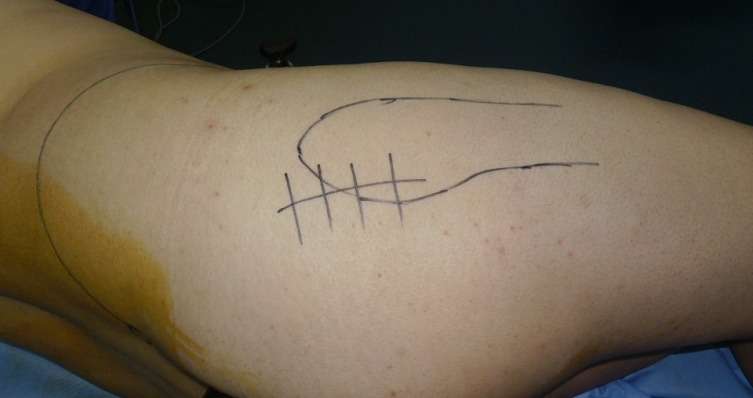
The mini-invasive postero-lateral approach

 Considering these arguments, it is a natural step to combine the short femoral stem prosthesis with the mini-invasive approach, allowing the patient to benefit from both the advantages of conserving bone stock, and those of protecting the soft tissues. 

**Fig. 3:**
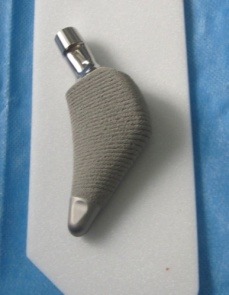
The Proxima prosthesis, with a short femoral stem

 From our point of view, only through documentation, exploration and research may we can gain enough knowledge to evaluate the clinical consequences of the long-term use of total hip arthroplasty through the mini-invasive method. Our aim is to establish a context in which to compare the functional results of this procedure with those of the classical procedure. 

## Case Presentation

- A. B., 35 years old, male; 

 - Diagnosis at admittance: 

 POST-TRAUMATIC LEFT HIP ARTHROSIS WITH FUNCTIONAL IMPAIRMENT AND 2 CM LIMP SHORTENING. 

 ROAD ACCIDENT POLYTRAUMA (MOTORCYCLE DRIVER) 24 MONTHS AGO. 

 MULTIPLE FRAGMENT FRACTURE OF THE ILIAC WING, ISCHION, ACETABULUM AND HEIPUBIS ON THE LEFT SIDE – OPERATED (OPEN REDUCTION AND OSTHEOSINTHESYS WITH PLATE AND SCREWS) – CONSOLIDATED. 

 MULTIPLE FRAGMENT INTRA-ARTICULAR FRACTURE OF THE DISTAL THIRD OF THE LEFT FEMUR WITH ARTICULAR – OPERATED- CONSOLIDATED.

 MULTIPLE FRAGMENT FRACTURE OF THE DISTAL THIRD OF THE LEFT TIBIA – OPERATED- CONSOLIDATED.

 FRACTURE OF THE MEDIAL THIRD OF THE LEFT TIBIAL AND FIBULA – OPERATED- CONSOLIDATED.

 No other known pathology.

**Fig. 4:**
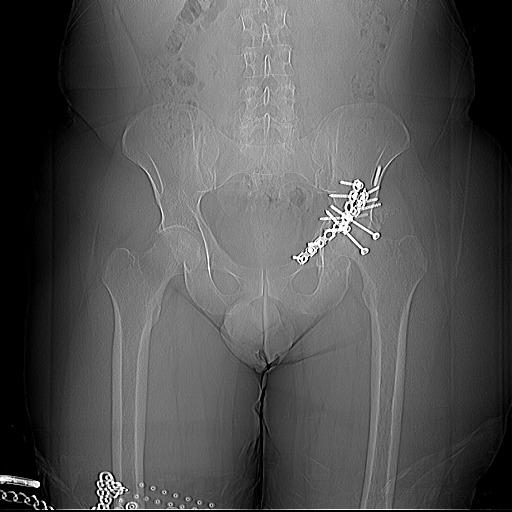
Preoperatory radiography

**Fig. 5:**
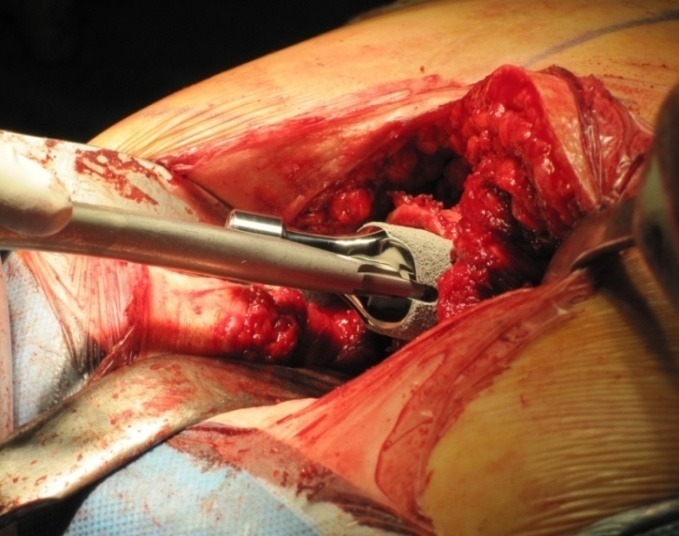
Impaction of the short stem femural prosthesis into the metaphysic

**Fig. 6:**
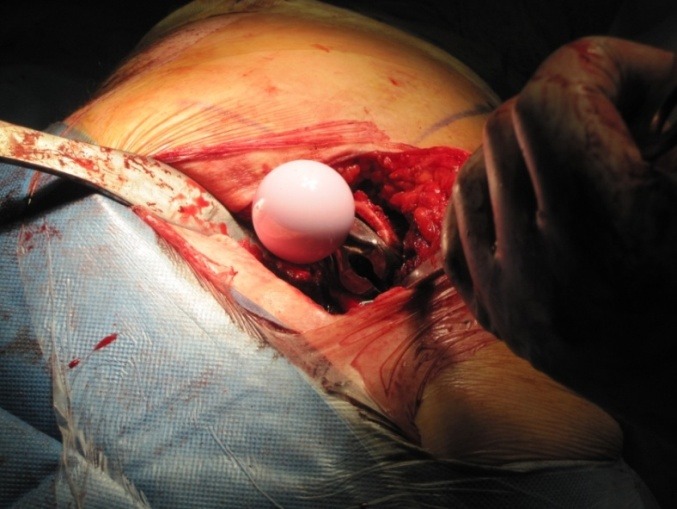
The ceramic prosthetic femoral head

**Fig. 7:**
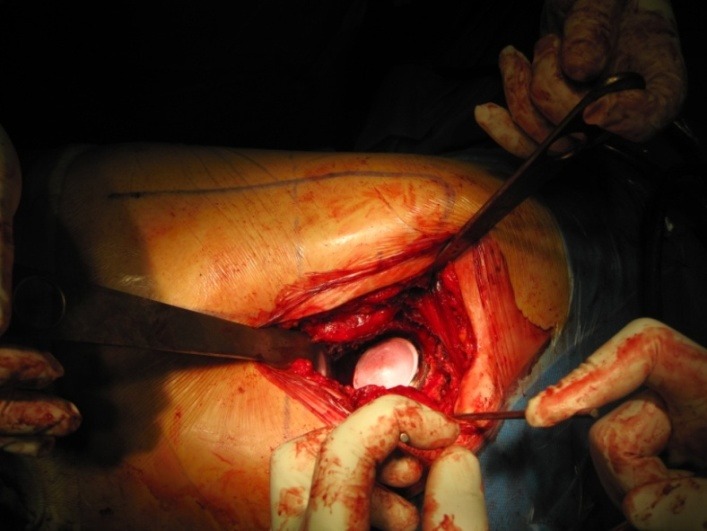
The acetabular cup with the ceramic insert

**Fig. 8:**
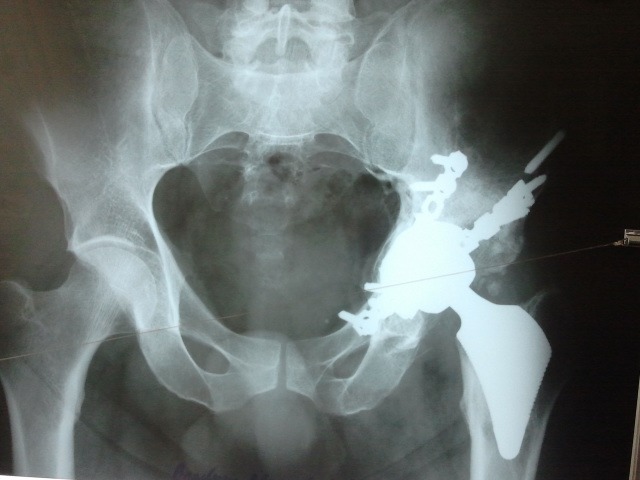
Postoperatory radiographic control

 Case particularities

-a national first in Romania, regarding total uncemented hip arthroplasty, with a short femoral stem, through a mini-invasive approach. 

 - a technically demanding case, due to the associated, consolidated pelvic fractures and the remaining ostheosynthesys material. 

 - the patient is young, active, but with a depleted bone stock, due to the long period of bed rest after his motorcycle accident.

 - the patient has a high potential of regaining his active lifestyle, including sports. 

## Results and Discussions

 Considering our observations up to this date, and analyzing the current day literature, we were able to differentiate a series of advantages and disadvantages regarding the mini-invasive short femoral stem total hip arthroplasty versus the classical method [**18-22**]: 

 Advantages: 

 1–conservation of femoral bone stock, through high neck osteotomies, preserving a large percentage of the neck, and preserving its natural anteversion; 

 2–a decrease in the cortical stress forces, thus significantly decreasing the percentage of thigh pain, one of the causes of painful hip prosthesis; 

 3–the possibility of implantation through mini-invasive techniques; 

 4–a longer expectancy of functionality, with the possibility of revision to a conventional femoral stem (a standardized procedure); 

 5–a viable alternative in important femoral deformities, in which a femoral prosthesis would be unsuitable; 

 Disadvantages: 

 1–routine intraoperatory radiographic control, the procedure having a learning curve; 

 2–if used in hip dysplasia, a much too anteversed implantation in the femoral neck would predispose to prosthesis displacement.

## Conclusions

Our current research suggests the superiority of surgical treatment through total hip arthroplasty with a short femoral stem, by using a minimally-invasive approach, versus the traditional method, based on the data showing a great number of significant advantages opposing the relatively minor disadvantages. 
